# Utilising a novel surveillance system to enhance field screening activities for the leishmaniases

**DOI:** 10.1016/j.mex.2020.101156

**Published:** 2020-11-27

**Authors:** Elina Panahi, Martin Shivas, Sonja Hall-Mendelin, Nina Kurucz, Penny A. Rudd, Rachel De Araujo, Eloise B. Skinner, Lorna Melville, Lara J. Herrero

**Affiliations:** a Institute for Glycomics, Griffith University, Southport, QLD 4222, Australia; b Mosquito Management, Brisbane City Council, Eagle Farm, QLD 4009, Australia; c Public Health Virology, Forensic and Scientific Services, Queensland Health, Coopers Plains, QLD 4108, Australia; d Medical Entomology, Centre for Disease Control, Top End Health Service, Casuarina NT 0811, Australia; e Berrimah Veterinary Laboratory, Department of Primary Industry and Resources, Berrimah, NT 0828, Australia; f Environmental Futures Research Institute, Griffith University, Southport, QLD 4222, Australia; g Redland Hospital, Metro South, Queensland Health, Cleveland, QLD 4163, Australia

**Keywords:** *Leishmania*, Biting insects, FTA^Ⓡ^ cards, Surveillance, Transmission

## Abstract

Over the last decade, an arbovirus surveillance system based on the preservation of nucleic acids (RNA/DNA) has been developed using Flinders Technology Associates (FTA^Ⓡ^) cards. Soaked in honey, FTA^Ⓡ^ cards are applied in the field to detect arboviruses expectorated during mosquito sugar feeding. This technique has been shown to be inexpensive and efficient, and the implementation of this system for detecting parasites could be of international importance. As *Leishmania* parasites are highly prevalent in developing countries, FTA^Ⓡ^ cards may offer an alternative inexpensive tool to enhance field surveillance activities for leishmaniasis. The simple approach of applying the cards in programs can substitute the necessary extensive training of personnel. In our hands, *Leishmania macropodum* DNA was shown to be stable on FTA^Ⓡ^ cards during a 10-week time course, supporting their suitability for projects where direct access to laboratories is unobtainable and samples require storage prior to processing. This method may benefit programs in remote areas where accessibility to laboratory facilities are limited and samples need to be stored long-term.

• This study found that FTA cards could be a valuable tool in the surveillance of leishmaniasis.
• The method is based on the long-term preservation and detection of *Leishmania* DNA expectorated during insect sugar feeding.
• The application of FTA cards can preclude the need to screen large samples and analysis of insect populations to provide evidence of disease transmission.

## Graphical abstract

**Figure:**
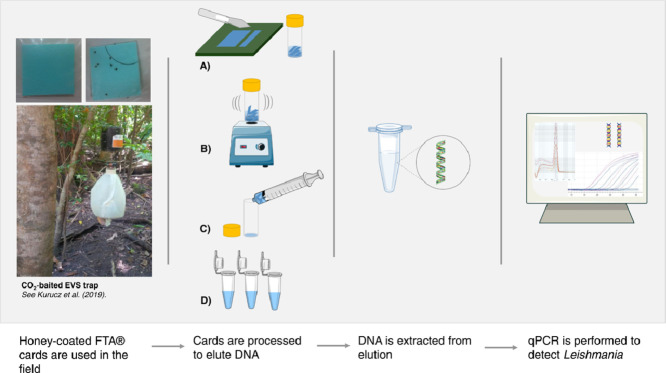

Specifications table

**Table:** 

Subject:	Immunology and Microbiology
More specific subject area:	*Disease surveillance programs*
Method name:	*Utilising FTA*^Ⓡ^*cards for Leishmania surveillance*
Name and reference of original method:	Hall-Mendelin, S., Hewitson, G.R., Genge, D., Burtonclay, P.J., De Jong, A.J., Pyke, A.T., van den Hurk, A.F., 2017. FTA Cards Facilitate Storage, Shipment, and Detection of Arboviruses in Infected Aedes aegypti Collected in Adult Mosquito Traps. Am. J. Trop. Med. Hyg. 96, 1241–1243. https://doi.org/10.4269/ajtmh.16–0981 Hall-Mendelin, S., Ritchie, S.A., Johansen, C.A., Zborowski, P., Cortis, G., Dandridge, S., Hall, R.A., van den Hurk, A.F., 2010. Exploiting mosquito sugar feeding to detect mosquito-borne pathogens. Proc. Natl. Acad. Sci. 107, 11,255–11,259. https://doi.org/10.1073/pnas.1002040107 van den Hurk, A.F., Hall-Mendelin, S., Townsend, M., Kurucz, N., Edwards, J., Ehlers, G., Rodwell, C., Moore, F.A., McMahon, J.L., Northill, J.A., Simmons, R.J., Cortis, G., Melville, L., Whelan, P.I., Ritchie, S.A., 2014. Applications of a Sugar-Based Surveillance System to Track Arboviruses in Wild Mosquito Populations. Vector-Borne Zoonotic Dis. 14, 66–73. https://doi.org/10.1089/vbz.2013.1373
Resource availability:	Whatman Flinders Technology Associates (FTA^Ⓡ^) cards (Purchase i.e. with Interpath).

## Method details

FTA^Ⓡ^ cards are coated in honey to feed biting insects and collect their saliva. Cards can then be processed to detect insect-borne pathogens by preserving nucleic acid (DNA and RNA) [1]. Additionally, blue dye is added to the honey, to provide evidence of feeding on inspection of insects. If this is not required, then omit the blue dye. Alternative sugar sources such as glucose, fructose, trehalose, etc. could potentially substitute honey however, they have not been trialled in this study.

### FTA^Ⓡ^ card preparation

The preparation does not have to be conducted under sterile conditions, as the cards will be used in field settings. It is important to wear laboratory gloves when handling FTA^Ⓡ^ cards to avoid nucleic acid contamination.

*Materials needed*

• Parafilm (or similar, to prevent cards’ from sticking to surfaces)
• FTA^Ⓡ^ Classic Card, 4 Sample Areas Per Card (Cat#WB120205, Interpath Australia)
• Disposable Pasteur pipettes
• Wooden applicator stick
• 100% pure honey
• Blue food dye
• 50 mL tubes
• Plastic resealable bags
• Silica beads

1. In a 50 mL tube, mix honey and blue dye to achieve a desired colour. Ensure that the final volume is sufficient to thoroughly coat the FTA^Ⓡ^ card. Start with a ratio of 80:1 (honey: dye) and mix by inverting several times or by using a wooden applicator stick to ensure a complete even consistency.
2. While wearing gloves, FTA^Ⓡ^ cards are cut into required sizes (Fig. 1). We have used 2.5 cm × 2.5 cm cards, and the following volumes for processing cards described in this protocol are adequate for this format.

**Fig. 1:**
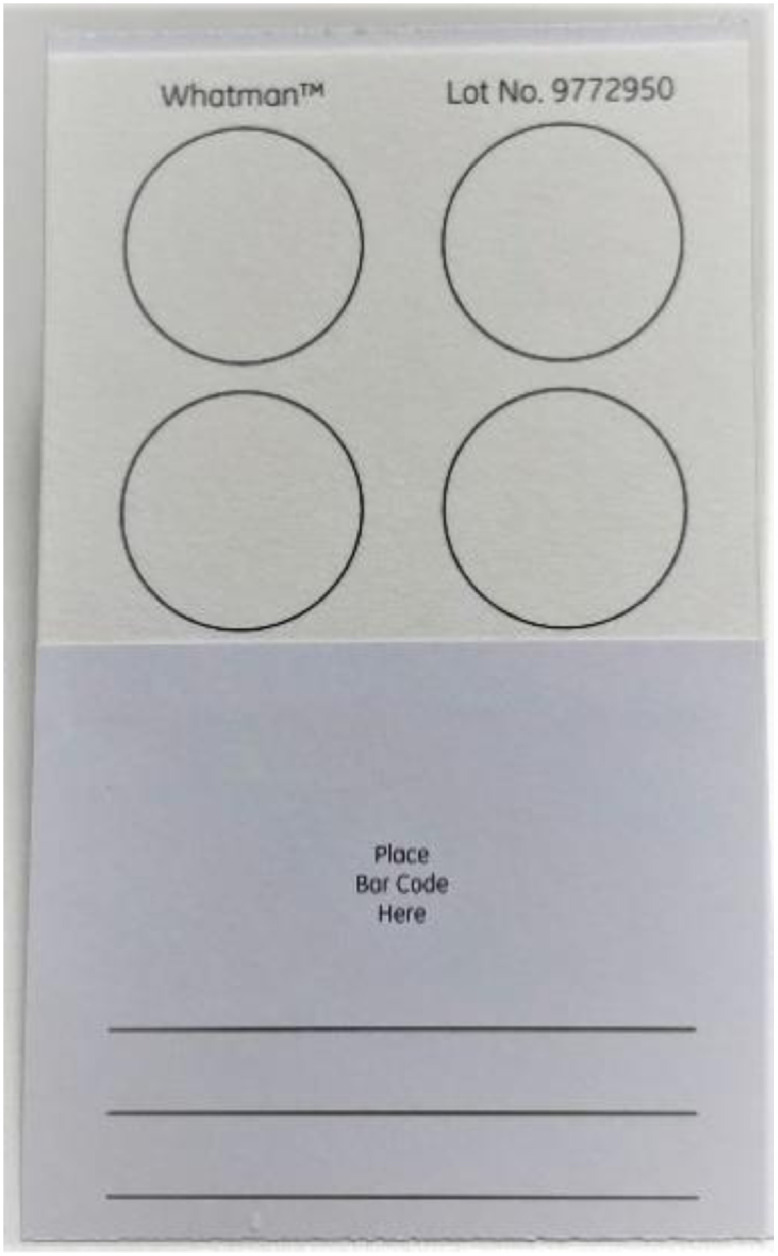
Classic FTA^Ⓡ^ card with four sample areas. Cards are designed to preserve nucleic acids. While the cards have pre-printed sample areas, the cards can be cut into any preferred angles and/or sizes.
3. Ensure to coat cards with honey 24–48 h prior to use allowing the honey to absorb into the cards evenly. Place cards onto parafilm and with a disposable Pasteur pipette add 0.5–1 mL of honey onto the cards and use the pipette to distribute the honey (Fig. 2). If the honey mix is too viscous for the Pasteur pipette, then try cutting the pipette tip for easier coating.

**Fig. 2:**
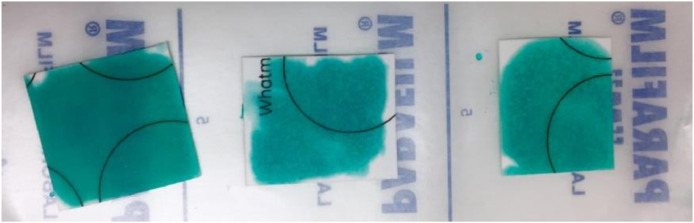
Honey-coated FTA^Ⓡ^ cards kept at room temperature. If cards are used in a location where humidity is high (> 90%) leave coated cards to dry for 48–72 h.
4. FTA^Ⓡ^ cards can be used with appropriate catch containers, by converting these to hold FTA^Ⓡ^ cards, as previously described [2, 3].
5. For FTA^Ⓡ^ cards storage (after field-collection), keep individual cards in plastic resealable bags with 2 – 4 silica beads. The silica beads will help to absorb humidity and to keep cards dry.

### DNA elution from FTA^Ⓡ^ cards

*Materials needed*

• 5 mL tubes
• 1.5 mL microcentrifuge tubes
• 20 mL plastic syringe (no needle)
• Sterile blades (or similar cutting instruments; i.e. sterilised scissors)
• Cutting matt (or alternative base)
• Sterile forceps (or disposable forceps)
• Molecular grade water (Nuclease –free Water, Cat#W4502, Sigma-Aldrich Australia)
• Milli Q water
• 10% bleach
• 80% ethanol
• Paper towels
• Vortex Mixer
• Ice

1. In a 5 mL tube, add 1 mL molecular grade water. If your cards are larger than 2.5 cm × 2.5 cm, then adjust the volume of water.
2. To elute DNA from FTA^Ⓡ^ cards, cut the cards into strips with either a sterile scalpel or sterilised scissors (one for each sample) and transfer them into the 5 mL tube from step 1 (Fig. 3). To avoid DNA degradation, keep the tube on ice.

**Fig. 3:**
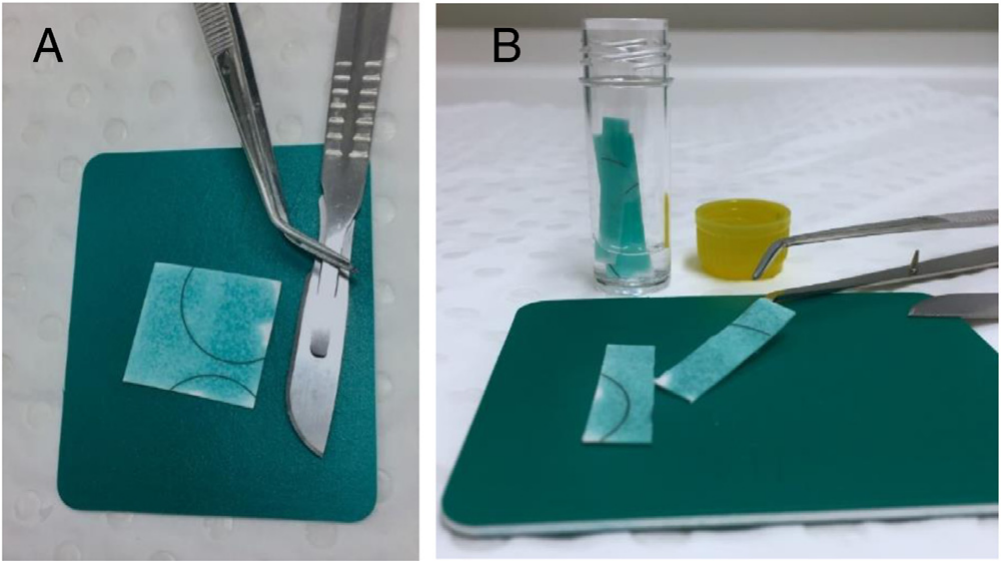
Set up of DNA elution process from FTA^Ⓡ^ cards. A) Sterile instruments are used to cut cards into smaller strips for easier elution of DNA. B) Strips are transferred into 5 mL tubes containing 1 mL molecular grade water and kept on ice. The volume added to the 5 mL tube will depend on the size of the card. For cards of the size 2.5 cm × 2.5 cm, a volume of 1 mL is suitable. Adjust the volume hereafter. It is important to remember that DNA extraction uses 200 µL of elution and the final volume should not be less than this.
3. Fresh instruments are used between each card (i.e. sterile blade, sterilised scissors and disposable forceps) to avoid cross-contamination. Alternatively, wash instruments in 10% bleach, followed by rinsing in Milli Q water and finally 80% ethanol. Dry off with tissue paper.
4. To release the DNA from the matrix of the FTA^Ⓡ^ cards, the tubes are vortexed for 10 – 20 s every 5 min for a total of 20 min (Fig. 4A). Between vortexing, leave the tubes on ice.

**Fig. 4:**
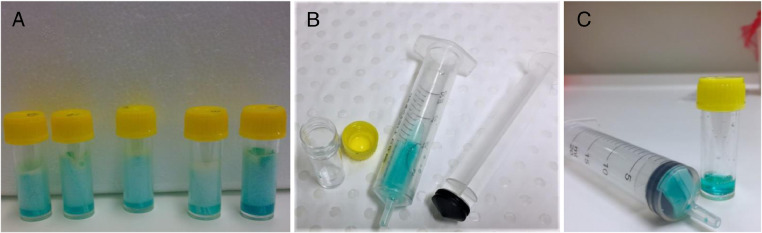
Process of releasing DNA from matrix of FTA^Ⓡ^ cards to final elution.
5. The strips and suspension are separated using a 20 mL syringe (Fig. 4B). A trick for easy elution is to remove the plunger from the syringe and pour in suspension of elution plus FTA^Ⓡ^ card strips into the syringe, then insert the plunger. Keep syringe horizontal when pouring to avoid spilling the suspension.
6. The suspension, now containing the DNA, is pressed into the same (or a new) 5 mL tube (Fig. 4C). Discard strips and syringe. Aliquot the suspension into 200 µL volume and store at –80 °C until further use.
7. Aliquots can be used for DNA extraction using commercially available kits following the manufacturer's protocol for purification of total DNA.

### Extracting DNA from FTA^Ⓡ^ cards

Whereas this protocol was optimised with DNeasy^Ⓡ^ Blood & Tissue Kit (Qiagen), any comparable kit would likely be an adequate substitute.

*Materials needed*

• DNA kit (DNeasy^Ⓡ^ Blood & Tissue Kit, Qiagen, Cat#69,506)
• 1.5 mL microcentrifuge tube
• Phosphate Buffer Solution (PBS; pH = 7.4), (Cat#P4417, Sigma Australia)
• Centrifuge, set at room temperature

1. One aliquot (200 µL) is used for DNA extraction either immediately after DNA elution (preferable) or thawed from –80 °C storage.
2. When thawed, centrifuge the tube at room temperature for 7 min at 800 *× g*.
3. Discard the supernatant and resuspend the pellet in 200 µL Phosphate Buffer Solution (PBS; pH = 7.4)
4. Follow the manufacturer's instructions for DNA extraction from cell cultures.
5. Repeat the final elution step by running 200 µL of elution buffer through the column twice.

### Real time qPCR screening: detection of *Leishmania* DNA

For detection and quantification of *Leishmania* parasite DNA, we utilised a Taqman PCR protocol specific to *Leishmania* (*Mundinia*) *macropodum.* This protocol was adapted from that previously described by Dougall et al. [4]. Additionally, this protocol can be adopted for screening other *Leishmania* parasites using published species-specific primers. Bio-Rad reagents were used in this protocol, however any comparable PCR reagents would be suitable with the required optimisation.

Genomic *L. macropodum* DNA standards from cultivated promastigotes were included in every PCR run to quantify parasite load in samples. Standards were purified from cultured *L. macropodum* promastigotes and made up of serial dilutions 10^−1^ – 10^−7^ in Tris-EDTA buffer (Sigma-Aldrich, Australia). Moreover, *L. macropodum* DNA standards were used to determine the qPCR assay's limit of detection.

*Materials needed*

• Primers:
  Forward (F), 5′-AAACTTCCGGAACCTGTCGT-3′ (Sigma, Australia)
  Reverse (R), 5′-GTAGGCACCCGAAGAGACC-3 (Sigma, Australia)
• Taqman probe 5′d FAM-CCGGCAAGATTTTGGGAGCG-BHQ-1 3′ (Sigma, Australia)
• SsoAdvanced Universal Probes Supermix (Cat#1,725,280, Bio-Rad Laboratories, Australia)
• Molecular grade water (Nuclease –free Water, Cat#W4502, Sigma-Aldrich Australia)
• MgCl_2_ Solution for PCR (Cat#1,708,872, Bio-Rad Laboratories, Australia)
• Genomic *Leishmania* DNA standards made up of serial dilutions 10^−1^ – 10^−7^
• Negative controls: DNA extraction from a honey-coated FTA ^Ⓡ^ card (not used in the field), and elution buffer (from DNA extraction kit)
• 1.5 mL amber microcentrifuge tubes
• Hard-Shell^Ⓡ^ 96-Well PCR Plates, low profile, thin wall, skirted, white/clear (Cat#HSP9601, Bio-Rad Laboratories, Australia)
• Microseal ‘B’ PCR Plate Sealing Film, adhesive, optical (Cat# MSB1001, Bio-Rad Laboratories, Australia)
• CFX96 Real Time System (C1000 Thermal Cycler) (Bio-Rad Laboratories, Australia)
• Microcentrifuge rack
• Plate centrifuge, 4 °C

1. Prepare working stocks of probe and primers in 1.5 mL amber microcentrifuge tubes
2. Prepare a 10 µM primermix (*F* + *R*) in an amber 1.5 mL microcentrifuge tube. Ensure to vortex followed by a quick spin to mix reagents evenly.
3. Each PCR reactions are made in a 10 µL reaction of 1 × SsoAdvanced™ universal probe Supermix, 6 mM MgCl_2_, 0.3 µM of primers, 0.05 µM Taqman probe and 2 µL of DNA template (Table 1). All reagents are kept cold.

**Table 1:** qPCR set up. All components must be kept cold. After thawing, ensure to vortex each component followed by a quick spin.

Components	Volume per 10 µl	Final concentration
SSOADVANCED UNIVERSAL PROBE SUPERMIX	5 µl	1x
PRIMERMIX (*F* + *R*)	0.3 µl	0.3 uM
TAGMAN PROBE	0.1 µl	0.05 uM
MGCL2	1.2 µl	6 mM
H20	1.40 µl	–
DNA (STANDARDS)	2 µl	–
TOTAL VOLUME	10.00 µl	**–**
4. Prepare a master mix in a 1.5 mL amber microcentrifuge tube of the reagents mentioned in step 3 and shown in Table 1 (do not add DNA template to master mix). Prepare enough master mix for all reactions (unknown and control samples) in duplicate.
5. Add 8 µL of the master mix to appropriate number of wells.
6. In each PCR test include a *no template control* (*NTC;* master mix only) as well as negative (honey-coated FTA^Ⓡ^ card and/or elution buffer) and positive (genomic DNA standards) controls.
7. Add 2 µL DNA template, controls and DNA standards in duplicate wells.
8. Use an optical adhesive PCR compatible to cover the PCR plate. Use a plate centrifuge to remove any bubbles and ensure the centrifuge is set at 4 °C.
9. The PCR cycling conditions are set as followed: 2 min at 95 °C followed by 35 cycles of 15 s at 95 °C and 40 s at 66 °C with the CFX96 Real Time System (C1000 Thermal Cycler; Bio-Rad Laboratories).

## Supplementary material and/or additional information

**Fig. 5:**
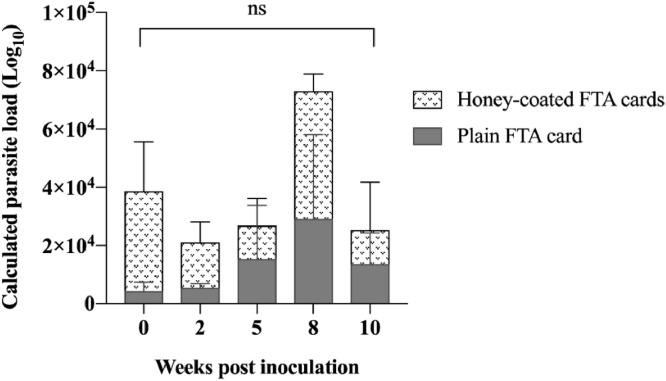
Evaluation of *L. macropodum* viability on FTA^Ⓡ^ cards and the effect of honey. To determine L. *macropodum* detection on honey-coated and plain FTA^Ⓡ^ cards, an experiment was designed over a 10-week time course. FTA^Ⓡ^ cards (2.5 cm × 2.5 cm) were inoculated with 10^6^ L. *macropodum* per card in triplicates and DNA was tested at five time points (week 0, 2, 5, 8 and 10). The limit of detection was 10^2^ parasites and the presence of honey had no significant effect of the detectability of *L. macropodum*.

From a long-term experiment assessing *Leishmania* DNA stability on FTA^Ⓡ^ cards, we showed the parasites were stable at room temperature over the entire course supporting their suitability for projects where long-term storage is unavoidable. Furthermore, the use of honey was not found to be associated with any interference of parasite load detection or the assay's sensitivity (Fig. 5).

## Declaration of Competing Interest

The Authors confirm that there are no conflicts of interest.
